# Relationship between Rad51 G135C and G172T Variants and the Susceptibility to Cancer: A Meta-Analysis Involving 54 Case-Control Studies

**DOI:** 10.1371/journal.pone.0087259

**Published:** 2014-01-27

**Authors:** Mengmeng Zhao, Pin Chen, Yanbin Dong, Xianji Zhu, Xilong Zhang

**Affiliations:** 1 Department of Respiratory Medicine, The First Affiliated Hospital of Nanjing Medical University, Nanjing, China; 2 Department of Neurosurgery, The First Affiliated Hospital of Nanjing Medical University, Nanjing, China; Baylor College of Medicine, United States of America

## Abstract

**Background:**

The associations between Rad51 gene polymorphisms (G135C and G172T) and risk of cancer have been investigated, but the results were inconclusive. To get a comprehensive evaluation of the association above, we performed a meta-analysis of published studies.

**Methods:**

A computerized search of PubMed, Embase and Web of Knowledge databases for all relevant studies was performed and the data were analyzed in a meta-analysis. The overall odds ratio (OR) with the 95% confidence interval (95% CI) was calculated to assess the strength of the association between Rad51 polymorphisms and cancer risk. Data were analyzed using fixed- or random-effects model when appropriate. Sensitivity analysis and publication bias test were also estimated.

**Results:**

Overall, a total of 54 case-control studies were included in the current meta-analysis, among which 42 studies with 19,142 cases and 20,363 controls for RAD51 G135C polymorphism and 12 studies with 6,646 cases and 6,783 controls for G172T polymorphism. For G135C polymorphism, the pooled results indicated that significantly increased risk was found in overall cancers (homozygote model: OR = 1.776, 95% CI = 1.288–2.449; allelic genetic model: OR = 1.169, 95% CI = 1.016–1.345; recessive model: OR = 1.946, 95% CI = 1.336–2.835), especially in breast cancer (homozygote model: OR = 1.498, 95% CI = 1.026–2.189; recessive model: OR = 1.732, 95% CI  =  1.170–2.562). For G172T polymorphism, a decreased cancer risk was observed in head and neck cancer (homozygote model: OR  =  0.621, 95% CI  =  0.460–0.837; allelic genetic model: OR  =  0.824, 95% CI  =  0.716–0.948; recessive model: OR  =  0.639, 95% CI = 0.488–0.837).

**Conclusions:**

Our results suggested that the Rad51 G135C polymorphism is a candidate for susceptibility to overall cancers, especially to breast cancer, and that the Rad51 G172T might play a protective role in the development of head and neck cancer.

## Introduction

Human cancer is still one of the leading causes of death worldwide, resulting in one of the most challenging global health issues confronted by mankind today. According to etiological studies, carcinogenesis of cancer is a complex, multistep and multifactor process, in which many genetic and environmental factors are involved. In recent years, it has become clear that individual variation in genetic backgrounds can lead to various consequences following the environmental exposure and may ultimately contribute to the cancer pathogenesis and progression [1]–[3].

DNA repair pathways are responsible for maintaining the genomic stability and integrity and play a pivotal role in protecting against genetic mutations [4]. DNA repair genes have been proposed as considerable factors in the prevention of genomic damage and continuously monitor chromosomes to correct injuries caused by exogenous agents such as ultraviolet light or cigarette smoke, and endogenous mutagens [5], [6]. Recent reports have indicated that genetic variation in DNA repair genes could cause altered DNA repair capacity, leading to accumulation of DNA damage, followed by programmed cell death or unregulated cell growth and may account, in part, for the cancer development [7].

Human RAD51, one of the key proteins for homologous recombination, is essential to meiotic and mitotic recombination and plays a crucial role in homologous recombination repair of DNA double-strand breaks [8]. It functions by forming nucleoprotein filaments on single stranded DNA, inducing homologous pairing and mediating strand exchange reactions between single and double stranded DNA during repair [9]. The RAD51 gene is located on chromosome 15q15.1 in humans and thought to participate in a common double-strand break repair pathway. In recent years, RAD51 gene polymorphisms have attracted wide-spread attention. Two commonly studied polymorphisms of RAD51 gene are G135C (rs1801320), a G to C transversion at position +135, and G172T (rs1801321), a G to T transversion in the 172 position. Both of them are located in the 5′ untranslated region and seem to be of functional relevance. These two polymorphisms were shown to affect mRNA stability or translational efficiency, leading to altered polypeptide product levels and altering the function of encoding RAD51 protein, and influenced the DNA repair capacity to some extent [10], [11].

In the past decade, a number of molecular epidemiological studies have been done to evaluate the association between RAD51 gene polymorphisms (G135C and G172T) and cancer risk in diverse populations, but the results remained controversial. Therefore, to derive a more precise estimation of the association between RAD51 G135C and G172T polymorphisms and cancer risk, a meta-analysis was performed. To the best of our knowledge, this is the most comprehensive meta-analysis regarding the RAD51 polymorphisms and cancer risk.

## Materials and Methods

### Search strategy and data extraction

All studies investigating the association between the RAD51 gene polymorphisms (G135C and G172T) and risk of cancer were identified by comprehensive computer-based searches of PubMed, Embase and Web of Knowledge databases (the last search update on August 25, 2013). The search was performed using various combinations of keywords like (“RAD51 gene” OR “RAD51 recombinase gene”) AND (“polymorphism” OR “variant” OR “variants”). The exact search is available on request from the authors. Additional studies were also identified by a hand search of all the references of retrieved articles. Our search was restricted to studies published in the English language.

### Inclusion and Exclusion criteria

Studies included in the current meta-analysis had to meet all the following criteria: (1) studies to investigate the associations between the polymorphisms of G135C or G172T in RAD51gene and risk of cancer; (2) an unrelated case-control or cohort design (3) sufficient data (genotype distributions for cases and controls) to calculate an odds ratio (OR) with its 95%CI; (4) studies published in English; (5) genotype distribution of control population consistent with Hardy-Weinberg Equilibrium (HWE). We did not consider abstracts or unpublished reports. Case reports, editorials, review articles, and letters were excluded. Articles were also excluded if they did not include a control population and did not determine genotype frequency. If studies with the same or overlapping data were published by the same authors, the study with the larger sample size was selected. The supporting PRISMA checklist is available as supporting information; see Checklist S1.

### Data extraction

Two authors extracted information from all eligible publications independently according to the inclusion criteria listed above. Disagreement was resolved by the evaluation of a third reviewer and discussion until a consensus was reached. The following characteristics were collected from each study: the first author, year of publication, country, patient ethnicity, cancer type, source of control groups (population- or hospital-based controls or mixed (composed of both population- and hospital-based controls)), and genotype frequencies in case and control groups. Meanwhile, we did not define any minimum number of cases or controls to be included in our meta-analysis.

### Statistical analysis

We first analyzed HWE in the controls for each study using goodness-of-fit test (chi-square or Fisher's exact test) and violation of HWE was determined by P<0.05. Crude odds ratios (ORs) with 95% confidence intervals (CIs) were used to assess the strength of association between the RAD51 gene polymorphisms and cancer susceptibility. The pooled ORs for RAD51 G135C polymorphism were performed under dominant model (CC+GC vs. GG), recessive model (CC vs. GG+GC), homozygote model (CC vs. GG) and allelic genetic model (C vs. G). C and G represent the minor and the major allele respectively. The same methods were applied to the analysis of the RAD51 G172T polymorphism. Stratified analyses were conducted with respect to ethnicity, cancer type and source of controls.

A χ^2^-based Q-test was performed to test the heterogeneity across the eligible comparisons, which is considered to be significant if P<0.05. The variation caused by heterogeneity was estimated by calculating the inconsistency index I^2^, with I^2^<25%, 25-75% and >75% representing low, moderate or high degrees of inconsistency, respectively [12]. The pooled OR was calculated by a fixed-effects model (the Mantel-Haenszel method) if the result of the Q test was P>0.05, which indicated that the between-study heterogeneity was not significant [13]. Otherwise, a random-effects model (the Der-Simonian and Laird method) was used [14]. Sensitivity analysis was carried out by removing each study at a time to evaluate the stability of the results under either genotypic models or the allelic model. Additionally, Begg's test and Egger's linear regression test by visual inspection of the funnel plot were carried out to address the potential publication bias and P<0.05 was considered as an indicator of significant publication bias [15].

All statistical analyses were performed using the STATA software (version 11; Stata Corporation, College Station, Texas). Two-sided P values less than 0.05 were considered significant.

## Results

### Studies included in the meta-analysis

**Figure 1:**
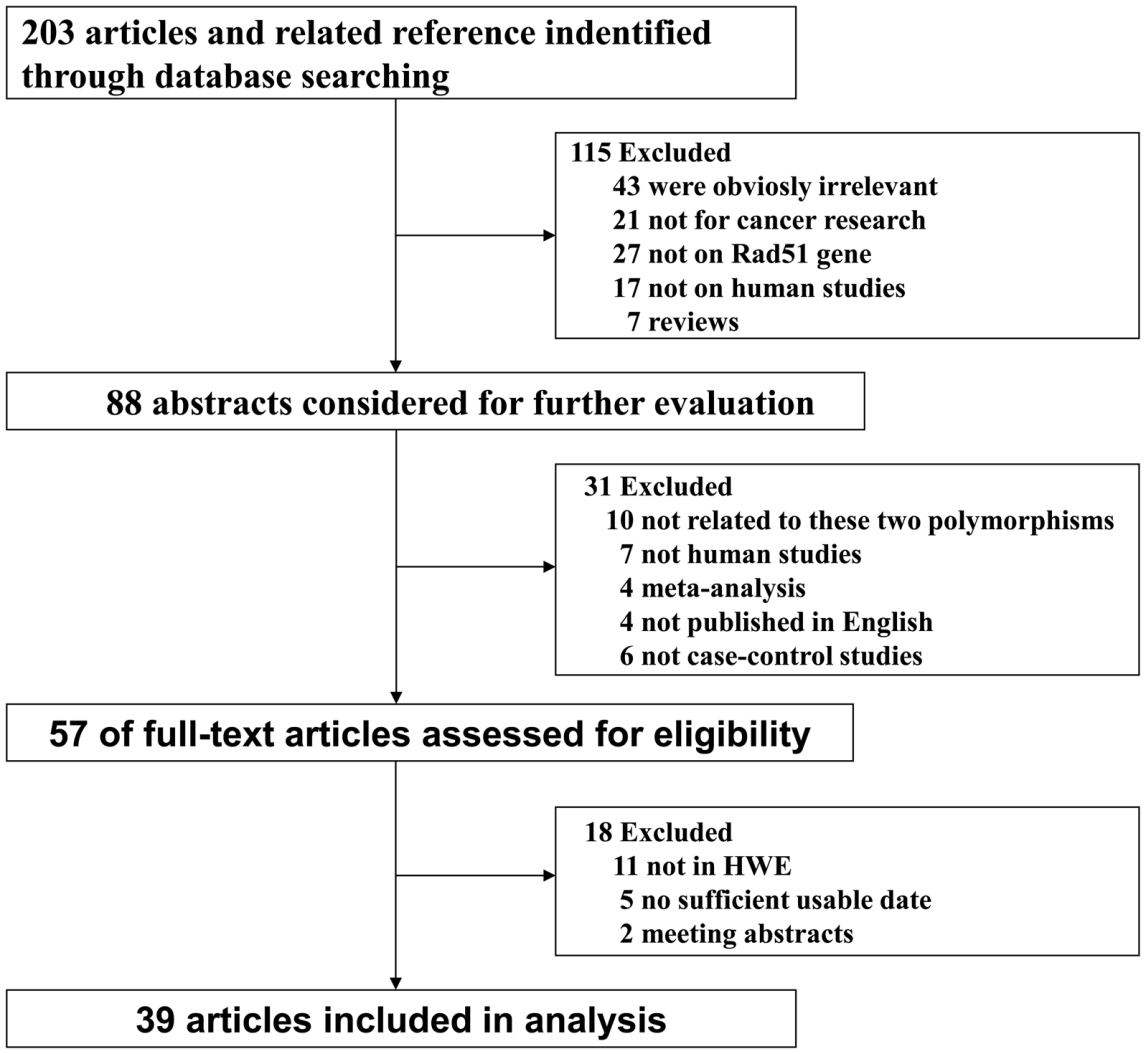
The flow diagram of the literature search and the study selection.

The initial literature search through PubMed, Embase and Web of Knowledge databases yielded 203 published articles after duplicates were removed. When reviewed by titles or abstracts, 115 records did not fulfill the inclusion criteria, leaving 88 potentially relevant studies that were reviewed in full-text. Among the remaining 88 articles, 10 were not concerned with G135C or G172T polymorphisms in RAD51gene, 7 were not human studies, 4 was not published in English, 6 were not case-control studies, 5 were no usable reported data, 2 were meeting abstracts, 4 were meta-analysis, and 11 were not in HWE; these publications were also excluded. Finally, a total of 54 case-control studies in 37 articles were identified in the current meta-analysis [16]–[54], among which 42 with 19142 cases and 20363 controls for RAD51 G135C polymorphism and 12 with 6646 cases and 6783 controls for G172T polymorphism. Genotype distributions in the controls of all selected studies are in agreement with HWE. The flow of study selection is shown in Figure 1, and the main characteristics of eligible studies were shown in Table 1 and Table 2.

**Table 1:** Characteristics of the studies included on Rad51 G135C polymorphism.

First author	Year	Country	Ethnicity	Cancer type	Source of control	Cases	Controls	GG	GC	CC	GG	GC	CC	HWE
Kuschel	2002	Germany	Caucasian	Breast	PB	2172	840	1904	255	13	722	116	2	0.23
Seedhouse	2003	UK	Caucasian	AML	PB	186	206	166	18	2	171	32	3	0.30
Wang	2004	USA	Caucasian	Glioma	Mixed	309	342	265	40	4	301	41	0	0.24
Webb (1)	2005	Australia	Australian, Caucasian	Breast	PB	1444	788	1221	212	11	676	104	8	0.08
Webb (2)	2005	Australia	Australian, Caucasian	Ovarian	PB	546	1126	457	85	4	971	145	10	0.08
Auranen (1)	2005	UK	Caucasian	Ovarian	PB	729	847	642	84	3	745	100	2	0.48
Auranen (2)	2005	UK	African	Ovarian	PB	326	419	270	52	4	357	61	1	0.24
Auranen (3)	2005	UK	Caucasian	Ovarian	PB	278	699	241	36	1	616	78	5	0.15
Auranen (4)	2005	UK	Caucasian	Ovarian	PB	296	840	266	29	1	722	116	2	0.23
Lee	2005	Korea	Asian	Breast	HB	782	587	611	143	28	450	123	14	0.11
Sliwinski	2005	Poland	Caucasian	Breast	HB	150	150	108	38	4	106	41	3	0.67
Hanna	2005	Poland	Caucasian	Breast	PB	100	106	31	40	29	21	48	37	0.45
Romanowicz	2006	Poland	Caucasian	Breast	PB	100	106	31	40	29	21	48	37	0.45
Tarasov	2006	Russia	Caucasian	Breast	PB	151	191	111	36	4	148	41	2	0.65
Rollinson	2006	UK	Caucasian	AML	HB	466	936	431	34	1	817	115	4	0.98
Antoniou	2007	UK	Caucasian, African	Breast	HB	4443	4069	3838	567	38	3485	565	19	0.44
Costa	2007	Portugal	Caucasian	Breast	PB	265	435	216	45	4	381	53	1	0.55
Lu	2006	USA	Caucasian	HNC	HB	716	719	624	91	1	622	96	1	0.17
Jakubowska (1)	2007	Poland	Caucasian	Breast	HB	258	258	210	48	0	188	68	2	0.12
Jakubowska (2)	2007	Poland	Caucasian	Ovarian	HB	127	127	104	23	0	89	37	1	0.17
D.Figueroa	2007	USA	Caucasian	Bladder	HB	1085	1032	932	147	6	909	116	7	0.12
Voso	2007	Italy	Caucasian	AML	HB	160	161	125	33	2	142	18	1	0.61
Jara	2007	Chile	Latin-American	Breast	PB	131	247	113	16	2	222	25	0	0.40
Werbrouck	2008	Belgium	Caucasian	HNC	HB	152	157	136	15	1	134	23	0	0.32
Synowiec	2008	Poland	Caucasian	Breast	PB	41	48	18	10	13	17	27	4	0.14
Jakubowska	2008	Poland	Caucasian	Breast	PB	1007	1069	785	207	15	822	232	15	0.76
Bhatla	2008	USA	Caucasian, African	AML	HB	452	646	374	73	5	555	85	6	0.18
Krupa	2009	Poland	Caucasian	Breast	PB	135	175	91	33	11	105	63	7	0.52
Dhillon	2009	Australia	Caucasian	Prostate	HB	116	132	97	18	1	119	13	0	0.55
Jara	2010	Chile	Latin-American	Breast	PB	267	500	232	33	2	441	58	1	0.53
Krupa	2010	Poland	Caucasian	Endometrial	PB	30	30	6	8	16	19	9	2	0.52
Romanowicz	2010	Poland	Caucasian	Breast	HB	220	220	141	69	10	157	58	5	0.89
Liu	2011	China	Asian	AML	HB	105	704	72	25	8	511	175	18	0.52
Gil	2011	Poland	Caucasian	Colorectal	HB	133	100	100	29	4	73	27	0	0.12
Hamdy	2011	Egypt	Caucasian	AML	PB	50	30	39	9	2	26	3	1	0.06
Romanowicz	2012	Poland	Caucasian	Endometrial	HB	230	236	40	25	165	59	132	45	0.06
Gresner	2012	Poland	Caucasian	HNC	PB	81	87	67	13	1	71	14	2	0.22
Zhang	2012	China	Asian	Cervical	HB	80	175	58	20	2	122	50	3	0.41
Mucha	2012	Poland	Caucasian	Colorectal	HB	200	200	161	34	5	157	37	6	0.05
Romanowicz	2012	Poland	Caucasian	Colorectal	HB	320	320	51	56	213	91	164	65	0.57
Romanowicz	2012	Poland	Caucasian	HNC	PB	253	253	174	69	10	190	58	5	0.82
Smolarz	2013	Poland	Caucasian	Breast	PB	50	50	8	8	34	14	26	10	0.74

PB: population based; HB: hospital based; HWE: Hardy-Weinberg equilibrium (significant at the 0.05 level)

AML: acute myelocytic leukemia; HNC: head and neck cancer.

**Table 2:** Characteristics of the studies included on Rad51 G172T polymorphism.

First author	Year	Country	Ethnicity	Cancer type	Source of control	Cases	Controls	GG	GC	TT	GG	GC	TT	HWE
Kuschel	2002	Germany	Caucasian	Breast	PB	2235	736	744	1061	430	226	371	139	0.54
Auranen(1)	2005	UK	Caucasian	Ovarian	PB	730	847	226	363	141	273	433	141	0.16
Auranen(2)	2005	UK	African	Ovarian	PB	321	412	119	145	57	149	189	74	0.30
Auranen(3)	2005	UK	Caucasian	Ovarian	PB	293	607	112	130	51	235	277	95	0.38
Auranen(4)	2005	UK	Caucasian	Ovarian	PB	300	736	94	157	49	226	371	139	0.54
Lee	2005	Korea	Asian	Breast	HB	784	591	721	54	9	533	54	4	0.05
Rollinson	2006	UK	Caucasian	AML	HB	469	940	144	225	100	331	445	164	0.49
Lu	2006	USA	American	HNC	HB	716	719	261	351	104	240	335	144	0.17
Silva	2009	Portugal	Caucasian	Breast	HB	288	548	94	139	55	168	275	105	0.69
Gresner	2012	Poland	Caucasian	HNC	PB	81	110	36	43	2	43	54	13	0.52
Romanowicz	2012	Poland	Caucasian	Colorectal	HB	320	320	81	150	89	84	142	94	0.05
Bastos	2009	Portugal.	Caucasian	Thyroid	HB	109	217	28	51	30	76	98	43	0.27

PB: population based; HB: hospital based; HWE: Hardy-Weinberg equilibrium (significant at the 0.05 level)

AML: acute myelocytic leukemia; HNC: head and neck cancer

### Meta-analysis result

The pooled results of meta-analysis for the association between RAD51 polymorphisms (G135C and G172T) and cancer susceptibility are shown in Tables 3 and Tables 4.

**Table 3:** Meta-analysis of the Rad51 G135C polymorphism on cancer risk.

G135C	CC vs GG			C vs G			Dominant model			Recessive model		
G/C	OR(95%CI) *P*	*Ph*	*I^2^*	OR(95%CI) *P*	*Ph*	*I^2^*	OR(95%CI) *P*	*Ph*	*I^2^*	OR(95%CI) *P*	*Ph*	*I^2^*
Overall	**1.776 (1.288,2.449)** * **<0.01**	<0.001	62	**1.169(1.016,1.345)** * **0.03**	<0.001	85.5	1.039(0.942,1.146)* 0.45	<0.001	59	**1.946(1.336,2.835)** * <0.01	<0.001	76.2
**Source of controls**												
PB	**1.638(1.063,2.523)** * 0.03	0.002	53	1.134(0.977,1.316)* 0.10	<0.001	69.9	1.031(0.901,1.181)* 0.65	0.003	52	**1.820(1.185,2.796)** * 0.01	<0.001	57.7
HB	**1.924(1.256,2.947)** * <001	0.001	59	1.166(0.909,1.495)* 0.23	<0.001	91.3	1.038(0.890,1.210)* 0.63	<0.001	68	**1.946(1.131,3.381)** * 0.02	<0.001	78.6
**Ethnicity**												
Caucasian	**1.793(1.179,2.727)** * 0.01	<0.001	67	1.182(0.968,1.444)* 0.10	<0.001	88.5	1.018(0.886,1.169)* 0.80	<0.001	66	**1.998(1.242,3.212)** * <0.01	<0.001	79.4
Asian	**1.797(1.073,3.010)** 0.03	0.361	1.9	1.056(0.880,1.267) 0.56	0.282	21.1	0.969(0.787,1.193) 0.77	0.53	0	**1.840(1.102,3.074)** 0.02	0.39	0
Mixed	**1.522(1.036,2.235)** 0.03	0.33	13	1.058(0.970,1.153) 0.20	0.408	2.3	1.041(0.949,1.141) 0.40	0.329	13	**1.513(1.031,2.221)** 0.04	0.309	15.8
**Cancer type**												
Breast	**1.498(1.026,2.189)** * 0.04	0.009	51	1.053(0.926,1.198)* 0.43	<0.001	65	0.966(0.859,1.087)* 0.57	0.026	44	**1.732(1.170,2.562)** * 0.01	0.001	58.7
Ovarian	1.135(0.869,2.823) 0.72	0.609	0	1.005(0.872,1.158) 0.94	0.071	50.8	1.000(0.861,1.163) 0.99	0.056	54	1.129(0.561,2.273) 0.73	0.622	0
AML	1.567(0.869,2.823) 0.14	0.454	0	1.074(0.719,1.605)* 0.73	<0.001	77.4	1.052(0.686, 1.614)* 0.82	0.001	76	1.557(0.865,2.803) 0.14	0.475	0
HNC	1.690(0.708,4.036) 0.24	0.718	0	1.050(0.858,1.284) 0.64	0.253	26.5	1.025(0.825,1.274) 0.82	0.285	21	1.628(0.683,3.879) 0.27	0.738	0
Colorectal	2.887(0.615,13.555)* 0.18	0.01	78	1.512(0.546,4.185)* 0.43	<0.001	95.1	1.212(0.654,2.245)* 0.54	0.007	80	3.356(0.580,19.397)* 0.18	0.002	83.7

P-values for ORs; *Ph* values of Q-test for heterogeneity test; I^2^ refers to the proportion of total variation owing to between-study heterogeneity

Bold data represent the positive results.

PB: population based; HB: hospital based; AML: acute myelocytic leukemia; HNC: head and neck cancer

* Random-effects model was used when *Ph* value for heterogeneity test <0.05; otherwise, fix-effects model was used.

**Table 4:** Meta-analysis of the Rad51 G172T polymorphism on cancer risk.

G172T	TT vs GG			T vs G			Dominant model			Recessive model		
G/T	OR (95%CI) *P*	*Ph*	I^2^	OR (95%CI) *P*	*Ph*	I^2^	OR (95%CI) *P*	*Ph*	I^2^	OR (95%CI) *P*	*Ph*	I^2^
Overall	1.014(0.852,1.206)* 0.88	0.016	53	0.993(0.941,1.048) 0.80	0.05	44.1	0.980(0.906,1.061) 0.62	0.414	3.1	1.011(0.872,1.173)*0.88	0.025	49.9
**Source of controls**												
PB	0.991(0.857,1.146) 0.91	0.19	33	0.990(0.922,1.062) 0.77	0.457	0	0.961(0.866,1.067) 0.46	0.834	0	1.025(0.901,1.166) 0.71	0.172	35.3
HB	1.087(0.783,1.508)* 0.62	0.007	69	1.014(0.871,1.180) 0.86	0.011	66.6	1.006(0.892,1.135) 0.92	0.111	44.2	1.035(0.791,1.353)* 0.80	0.015	64.3
**Ethnicity**												
Caucasian	1.066(0.943,1.206) 0.31	0.07	45	1.027(0.966,1.091) 0.39	0.116	37.9	1.014(0.926,1.110) 0.76	0.373	7.5	1.067(0.957,1.188) 0.24	0.15	33.5
Mixed	**0.756(0.590,0.968)** 0.03	0.161	49	**0.882(0.781,0.996)** 0.04	0.241	27.3	0.902(0.757,1.077) 0.25	0.613	0	**0.772(0.617,0.965)** 0.02	0.12	58.7
**Cancer type**												
Breast	0.957(0.779,1.175) 0.67	0.647	0	0.949(0.860,1.048) 0.30	0.875	0	0.880(0.763,1.016) 0.08	0.863	0	1.030(0.859,1.234) 0.75	0.693	0
Ovarian	1.059(0.881,1.273) 0.54	0.528	0	1.023(0.936,1.119) 0.61	0.642	0	1.014(0.888,1.157) 0.84	0.945	0	1.059(0.899,1.247) 0.50	0.424	0
HNC	**0.621(0.460,0.837)** <0.01	0.111	61	**0.824(0.716,0.948)** 0.01	0.502	0	0.864(0.705,1.060) 0.16	0.788	0	**0.639(0.488,0.837)** <0.01	0.103	62.4

P-values for ORs; *Ph* values of Q-test for heterogeneity test; I^2^ refers to the proportion of total variation owing to between-study heterogeneity

Bold data represent the positive results.

PB: population based; HB: hospital based; AML: acute myelocytic leukemia; HNC: head and neck cancer

* Random-effects model was used when *Ph* value for heterogeneity test <0.05; otherwise, fix-effects model was used.

**Figure 2:**
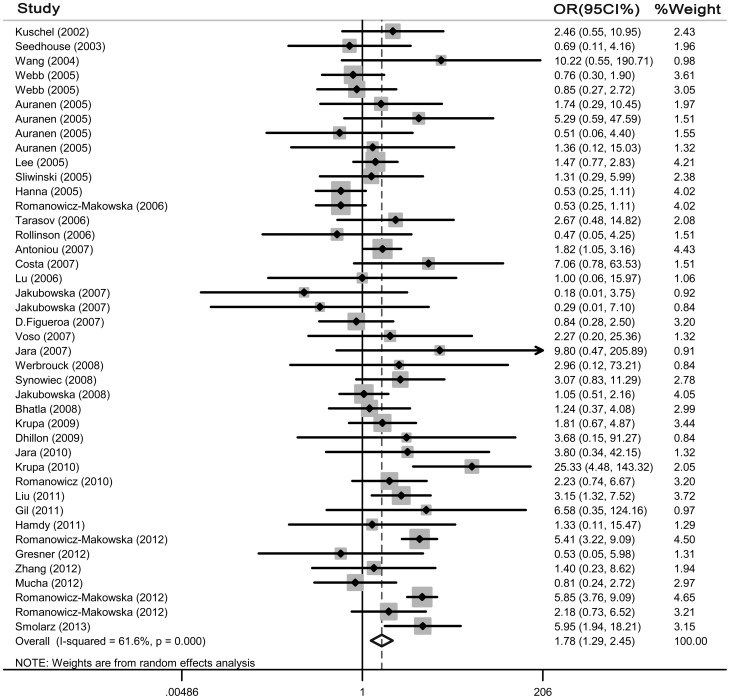
Forest plot for association of Rad51 G135C polymorphism and cancer risk (homozygote model, CC vs. GG).

As for G135C polymorphism, a total of 42 case-control studies in 37 publications with 19,142 cases and 20,363 controls were identified. Overall, significantly elevated cancer risk was found in all genetic models (homozygote model: OR = 1.776, 95% CI = 1.288–2.449, Figure 2; allelic genetic model: OR = 1.169, 95% CI = 1.016–1.345; recessive model: OR = 1.946, 95% CI = 1.336–2.835) except in dominant model (OR = 1.039, 95% CI = 0.942–1.146). The heterogeneity was significant in all genetic models and the detailed data are shown in Table 3. These eligible studies were analyzed by stratified analysis. In the stratified analysis of the effect of cancer types, a significant association was found for breast cancer (homozygote model: OR = 1.498, 95% CI = 1.026 –2.189; recessive model: OR = 1.732, 95% CI = 1.170–2.562). However, no significant association with cancer risk was demonstrated in overall population with ovarian cancer, colorectal cancer, acute myelocytic leukemia as well as head and neck cancers. As for ethnicity, our results showed G135C polymorphism was associated with increased risk of cancer among all populations under homozygote model and recessive model. When stratified based on source of controls, significantly increased risks were also observed in both population-based control subgroups and hospital-based control subgroups (Table 3).

**Figure 3:**
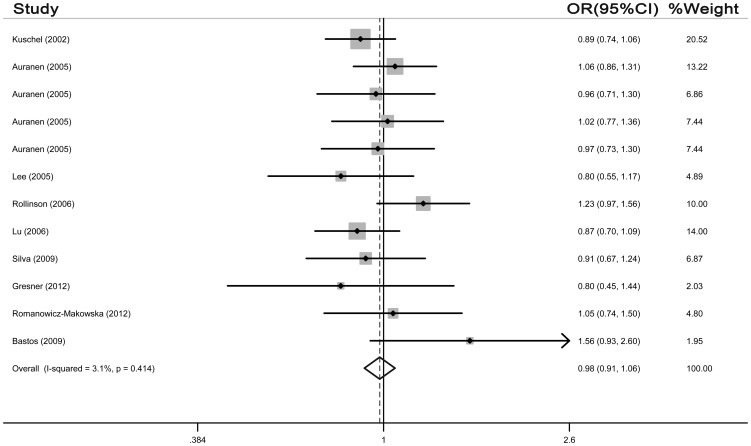
Forest plot for association of Rad51 G172T polymorphism and cancer risk (dominant model, TT+GT vs. GG).

With respect to G172T polymorphism, a total of 12 case-control studies in 9 publications with 6,646 cases and 6,783 controls were selected. As shown in Table 4, the pooled results revealed no significant associations between G172T polymorphism and cancer susceptibility in all genetic models (homozygote model: OR = 1.014, 95% CI = 0.872–1.173; dominant model: OR = 0.980, 95% CI = 0.906–1.061, Figure 3; recessive model: OR = 1.011, 95% CI = 1.241–14.879; allelic genetic model: OR = 0.993, 95% CI = 0.941–1.048). The heterogeneity was significant in all genetic models except for dominant model (P = 0.414). We also analyzed these eligible studies by stratified analysis. As we divided the studies by cancer type, the result suggested that a decreased cancer risk was found in head and neck cancers (homozygote model: OR = 0.621, 95% CI = 0.460–0.837; allelic genetic model: OR = 0.824, 95% CI = 0.716–0.948; recessive genetic model: OR = 0.639, 95% CI = 0.488–0.837) Nevertheless, we did not find significant association between G172T polymorphism and breast cancer and ovarian cancer. When stratified according to ethnicity, the result showed no evidence that the G172T polymorphism was significantly associated with an increased cancer risk in Caucasian populations. In the subgroup analysis by source of controls, no significant association with cancer risk was observed in both population-based and hospital-based control subgroups (Table 4).

### Sensitive analysis

Given that the significant between-study heterogeneity for RAD51 G135C and G172T polymorphisms, the random-effect model was used to calculate the pooled results if the heterogeneity was significant. Meanwhile, we also performed a sensitivity analysis to assess the effects of each study on the pooled ORs by omission of individual studies. The sensitivity analysis showed that, for each polymorphism, no single study qualitatively changed the pooled ORs, suggesting that the results of this meta-analysis were statistically stable and reliable (Figure_S1 and Figure _S2).

### Publication bias diagnostics

**Figure 4:**
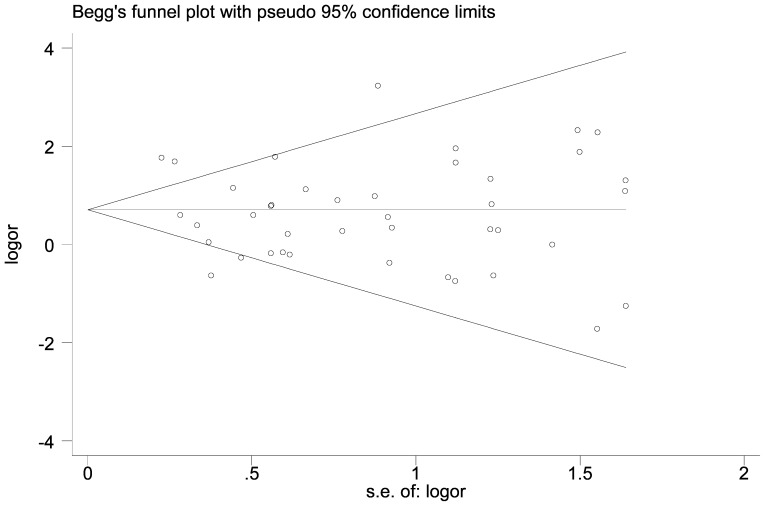
Begg's funnel plot for publication bias in studies on Rad51 G135C polymorphism and cancer (homozygote model, CC vs. GG).

**Figure 5:**
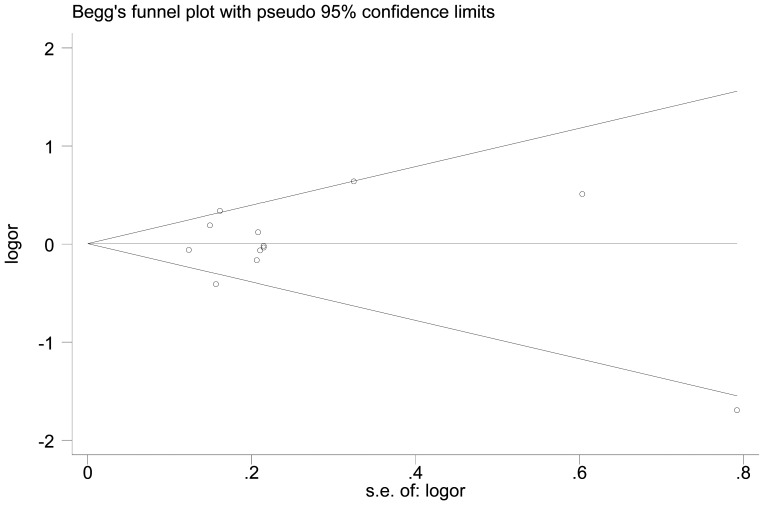
Begg's funnel plot for publication bias in studies on Rad51 G172T polymorphism and cancer (homozygote model, TT vs. GG).

We further identify the potential publication biases of literatures by Egger's test and funnel plot. In all studies, no funnel plot asymmetry was found. The results of the Egger's test for RAD51 G135C and G172T polymorphisms did not show any evidence of publication bias (t = −1.11, P = 0.275 for G135C under homozygote comparison model, Figure 4; t = −0.09, P = 0.929 for G172T under homozygote comparison model, Figure 5).

## Discussion

It is well reported that double-strand break damage is the most dangerous lesion observed in eukaryotic cells because it may cause cell death or constitute a serious threat to cell viability and genome stability. It has the potentiality to permanently arrest cell cycle progression and endanger cell survival [55]. Since DNA repair mechanisms are essential to preserve genomic stability and functionality, defects in DNA repair can result in the development of chromosomal aberrations which may lead to an increased susceptibility to cancer [4], [56], [57]. Homologous recombination and non-homologous end joining have been extensively studied as two distinct pathways in the repair of double-strand breaks in mammalian cells. Homologous recombination is a high-fidelity process that utilizes DNA sequence, a sister chromatid or homologous chromosome in close proximity to the break as a template [58]–[60]. In this repair process, an early procedure is the resection of the 3′ends of the DSBs to form single stranded tails that invade the intact homologous DNA double helix forming a Holliday junction [61], [62]. RAD51, a kind of ubiquitous strand exchange protein, is known to be a central enzyme involved in DNA double-strand break repair by homologous recombination. It could polymerize onto single-stranded DNA and searches for homology in a duplex donor DNA molecule, usually the sister chromatid [63]. Recent researches have suggested two common polymorphisms (G135C and G172T) located in the 5′ untranslated region seems to be of functional relevance. Furthermore, many functional studies revealed that these polymorphisms could affect mRNA stability or translational efficiency, resulting in changes in both polypeptide product levels and the function of encoding RAD51 protein, and thus influenced the DNA repair capacity to some extent [10], [11]. In addition, the association of Rad51 variants (G135C and G172T) and risk of cancer has been extensively investigated in different populations. However, the results of these studies were inconsistent. Therefore, we conducted a meta-analysis to summarize the effects of Rad51 variation on risk of cancer.

In this meta-analysis, 54 case-control studies (42 for G135C polymorphism, 12 for G172T polymorphism) were performed to provide the most comprehensive assessment of the relationship between RAD51 polymorphisms and cancer risk. For Rad51 G135C polymorphism, the C allele of G135C polymorphism had significant association with the cancer susceptibility for the homozygote model, allelic genetic model, and recessive genetic model in overall populations. Nevertheless, the results suggested that Rad51 G172T polymorphism was not associated with overall cancer risk when all studies were accumulated together. Considering the possible role of ethnic differences in genetic backgrounds, we performed subgroup analysis based on ethnicity. Consequently, significant association was found in both Caucasians and Asians for Rad51 G135C polymorphism but not for G172T polymorphism. When stratified by the source of controls, our results found evidence of an association between cancer risk and G135C polymorphism in both population-based and hospital-based controls, while no significant association was indicated in either population-based or hospital-based controls for G172T polymorphism. In the stratified analysis by cancer type, our results strongly indicated that Rad51 G135C polymorphism was associated with increased breast cancer risk while G172T polymorphism with decreased head and neck cancer risk.

Previous meta-analyses were carried out to assess the effect of Rad51 G135C polymorphism on either the risk of breast cancer or acute leukemia [64], [65]. Comparing with them, our study has some improvements. First, this is the first report not only to analyze two polymorphisms in Rad51 gene (G135C and G172T) and cancer risk in different cancer forms, but also to identify the G172T polymorphism as a risk factor for head and neck cancers. Second, we provided a more comprehensive data analysis by calculating four different genetic models and performing subgroup analysis based on ethnicity, cancer types and source of controls. Third, we excluded those studies in which genotype distributions in the controls were not in agreement with HWE because they could influence the results.

Heterogeneity between studies should be noted because it may affect the strengths of the meta-analysis. In the current meta-analysis, significance heterogeneity was observed for both Rad51 G135C and G172T polymorphisms. Thus, random-effect models were used if significant heterogeneity was identified. Meanwhile, to diminish the heterogeneity, we carried out subgroup analysis based on ethnicity, cancer types and source of controls. The results indicated that heterogeneity reduced or disappeared in subgroups. We also performed sensitivity analysis to ascertain the primary origin of the heterogeneity. The analysis showed that no single study materially altered the pooled ORs, suggesting that the results of this meta-analysis were statistically stable and reliable. The publication bias for the association between these two polymorphism and cancer risk was not observed.

Some limitations of the present meta-analysis should be taken into consideration when interpreting the results. First of all, only published studies and papers written in English were searched in this meta-analysis, some unpublished studies or studies written in other language that might also meet the inclusion criteria were overlooked. Second, in some studies, detailed information such as age and sex in case and control of different genotypes were not available, which limited further estimates to a certain extent. Third, the current meta-analysis did not consider gene-gene and gene-environment interactions due to the lack of sufficient data. Further studies are needed to evaluate the possible gene-gene and gene-environment interactions in the association between Rad51 gene polymorphism and susceptibility to cancer. Fourth, most of the patients in our study were Caucasians, which may limit the general application of our results. In spite of these, our present meta-analysis also had some advantages. First, analyzing two Rad51 gene polymorphisms with a total of 54 case-control studies has a much greater statistical power compared with any single study. Second, we excluded the studies in which genotype frequencies in controls were not in accordance with HWE, providing sufficient evidence for drawing safe conclusions about the association between the Rad51 polymorphisms and cancer risk. Third, the stability and credibility of the present meta-analysis was confirmed by the sensitivity analyses and publication biases analyses. Last, the findings highlight the association between Rad51 gene polymorphisms and cancer development and will provide directions for future research on molecular mechanism of cancer.

## Conclusions

Our investigations suggested that the Rad51 G135C polymorphism is a candidate for susceptibility to overall cancers, especially to breast cancer, and that the G172T polymorphism is significantly associated with decreased risk of head and neck cancers. Further studies are needed with large sample size and deeper evaluation about the effect of gene-gene and gene-environment interactions on the Rad51 polymorphisms and cancer risk.

## Supporting Information

Figure S1 **Sensitivity analysis of the summary OR of the association between Rad51 G135C polymorphism and cancer susceptibility in homozygote model.**
(TIF)

Figure S2 **Sensitivity analysis of the summary OR of the association between Rad51 G172T polymorphism and cancer susceptibility in homozygote model.**
(TIF)

Checklist S1 **PRISMA Checklist.**
(DOCX)
