# Supplementary figures and images for: Relationship between Rad51 G135C and G172T Variants and the Susceptibility to Cancer: A Meta-Analysis Involving 54 Case-Control Studies
Source: PLoS One. 2014 Jan 27;9(1):e87259. doi: 10.1371/journal.pone.0087259 (PMC3903631; doi:10.1371/journal.pone.0087259)

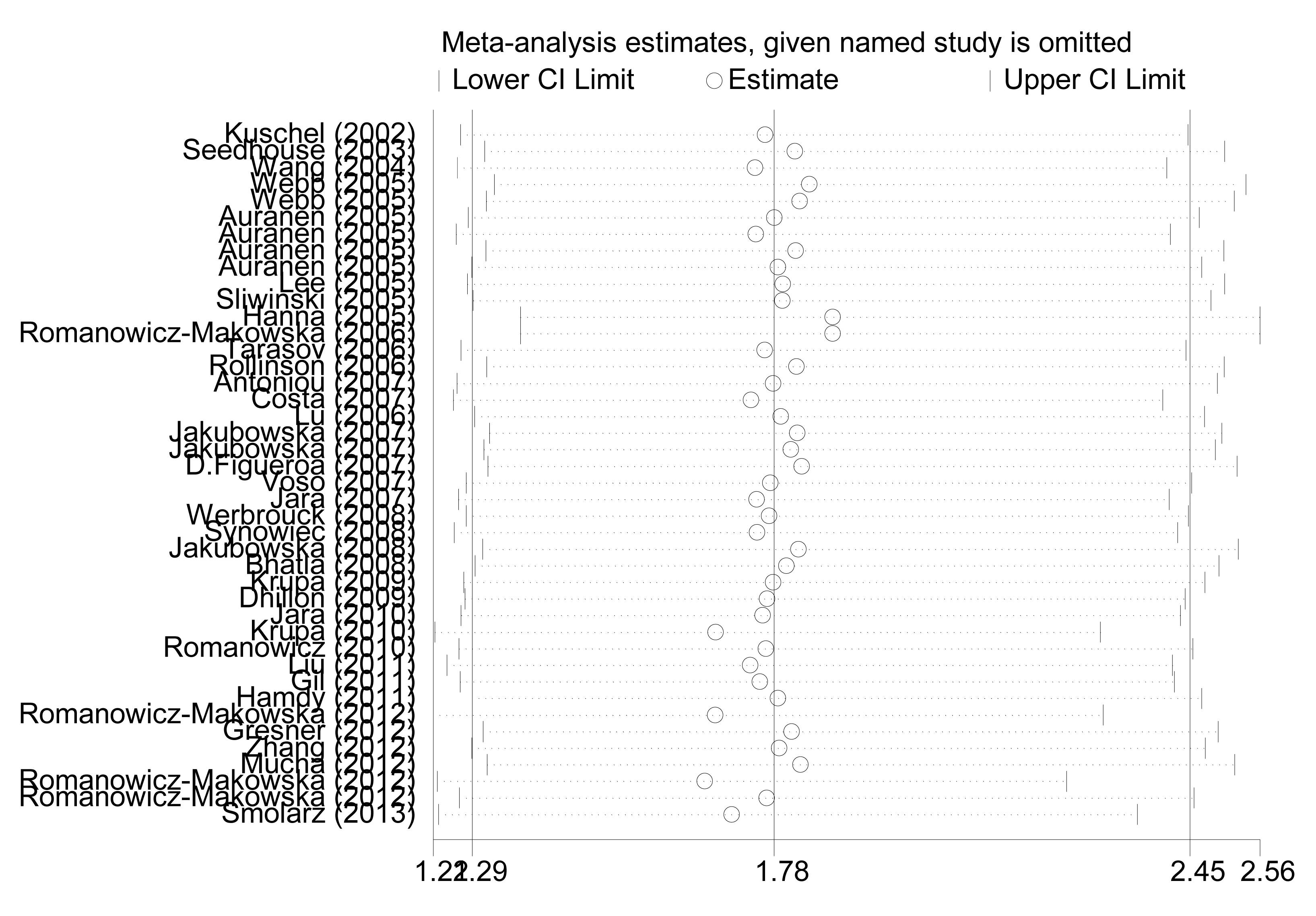

Supplement: Figure S1 — Sensitivity analysis of the summary OR of the association between Rad51 G135C polymorphism and cancer susceptibility in homozygote model. (TIF) [file pone.0087259.s001.tif]

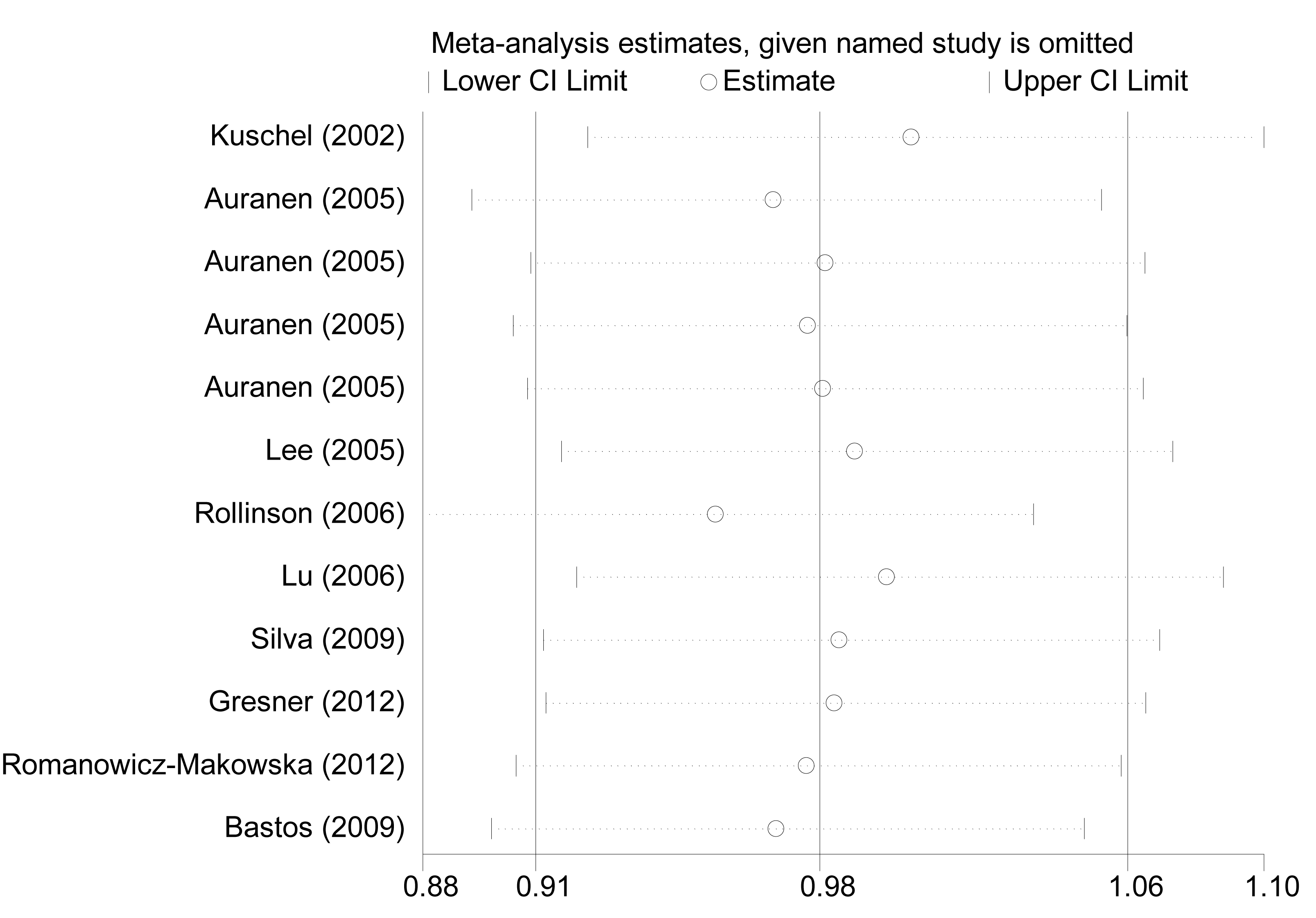

Supplement: Figure S2 — Sensitivity analysis of the summary OR of the association between Rad51 G172T polymorphism and cancer susceptibility in homozygote model. (TIF) [file pone.0087259.s002.tif]
